# Paraneoplastic pemphigus and Castleman’s disease: a case report and a revision of the literature

**DOI:** 10.1186/s13052-023-01442-7

**Published:** 2023-03-20

**Authors:** Mariangela Irrera, Elena Bozzola, Antonello Cardoni, Rita DeVito, Andrea Diociaiuti, Maya El Hachem, Katia Girardi, Alessandra Marchesi, Alberto Villani

**Affiliations:** 1 Pediatric Unit, IRCCS Bambino Gesù Children’s Hospital, Rome, Italy; 2 Unity of Pathology, IRCCS Bambino Gesù Children’s Hospital, Rome, Italy; 3 Dermatology Unit and Genodermatosis Unit, Genetics and Rare Diseases Research Division, IRCCS Bambino Gesù Children’s Hospital, Rome, Italy; 4 Department of Pediatric Hematology/Oncology and Cell and Gene Therapy, IRCCS Bambino Gesù Children’s Hospital, Rome, Italy

**Keywords:** Paraneoplastic pemphigus, Castelman’s disease, Child, Therapy, Case report

## Abstract

**Background:**

In literature, a few reports described an association between paraneoplastic pemphigus (PNP) and Castelman’s disease (CD), but no consensus have been proposed for the diagnostic-therapeutical approach. Aim of this study is to present a case report and explore the relationship between PNP and CD in pediatric patients, focusing on clinical manifestations, histopathological findings, treatment and outcome to find elements for an early diagnosis.

**Case presentation:**

We present the clinical case of a 13 years old girl with a challenging diagnosis of PNP and CD who underwent therapy at first with Rituximab and then with Siltuximab, for the control of symptoms.

**Conclusions:**

Reviewing literature, 20 clinical cases have been described in the pediatric age. Diagnosis may be challenging, requiring an average of 3 months (range from 3 weeks to 2 years). In all cases, the initial manifestations were mucocutaneous lesions, especially oral lesions with poor response to conventional treatment. Systemic symptoms may be present as well. Therapeutical approach is still discussed with no consensus. Almost all patients received corticosteroids with poor response. Other drugs including azathioprine, methotrexate, cyclosporine and monoclonal antibodies have been evaluated for the control of the disease. Further studies and experimental trials urge to define the diagnostic criteria and therapy protocol.

## Background

Paraneoplastic pemphigus (PNP) is a mucocutaneous autoimmune disorder, firstly described by Dr Anhalt in 1990 [1]. It is characterized by mucocutaneous manifestations associated with an underlying tumor that usually is suspected because of the refractoriness of mucocutaneous lesions to treatment.

The diagnosis is made by Mimouni’s criteria, which include:

1. Features of severe mucosal involvement with polymorphic cutaneous lesions
2. Detection of antiplakin autoantibodies
3. Histological findings of acantholysis or lichenoid or interface dermatitis
4. Presence of an underlying neoplasm especially lymphoproliferative malignancies [2].

The clinical picture is characterized by polymorphic mucocutaneous manifestations such as oral lesions, conjunctivitis, genital ulcers, skin eruption, so it may mimic other pathologies such as herpes simplex virus infection, Stevens-Johnson syndrome, lichen planus pemphigoids, lupus erythematosus, Behcet’s disease or other pemphigus. The clinical picture can be also characterized by a pulmonary epithelial involvement often leading to life-threatening bronchiolitis obliterans (BO).

In adults, the coexisting tumors are mostly non‐Hodgkin’s lymphoma (42%) and chronic lymphocytic leukemia (29%), while in paediatric patients PNP is most commonly associated with Castleman’s Disease (CD) [2, 3].

CD is a rare lymphoproliferative disorder that includes a group of heterogeneous hematologic disorders with a spectrum of characteristic, but nonspecific histopathologic features. This disease encompasses two entities, unicentric CD (UCD), which involves a single enlarged lymph node or region of lymph nodes, and multicentric CD (MCD) which involves multiple lymph node stations. MCD is in turn divided in idiopathic MCD (iMCD), POEMS (polyneuropathy, organomegaly, endocrinopathy, monoclonal plasma cell disorder, skin changes)—associated MDC (POEMS-MCD) and HHV-8 associated MCD (HHV8-MCD) [4]. When present, symptoms and signs are aspecific, such as linfoadenophaty, abdominal pain, anemia, fever, fatigue, weight loss and diagnosis is made by clinical picture and histological examination. It is unclear why CD is related to the pathogenesis of PNP. One hypothesis is that Castleman's tumors' proteins function as antigens and induce the production of autoantibodies cross-reacting with skin antigens. On the other side, secreted autoantibody from Castleman's tumors, which reacts against epidermal proteins, could be an essential factor in the pathogenesis of paraneoplastic pemphigus [5, 6].

Aim of this study is to present a case report and explore the relationship between PNP and CD in pediatric patients, focusing on clinical manifestations, histopathological findings, treatment and outcome to find elements for an early diagnosis.

## Case presentation

We report a clinical case and perform a literature review of PNP and CD in children between 1990 and 2022. The clinical manifestations, pathological findings, treatment and outcome are reported and analyzed.

This scoping review has been performed according to the PRISMA Extension guide for Scoping Reviews. An electronic search was undertaken on PubMed database on October 12th, 2022. To avoid missing results that may be of note for our revision study, constructing our search in PubMed, we used all of the important concepts from our basic clinical question, avoiding unnecessary filters. So, the terms “Castelman’s disease” and “Paraneoplastic Pemphigus” have been used as Mesh Terms.

Studies were identified as eligible for this scoping review if they met the following inclusion criteria:

- full-length articles or reviews pertaining to children and adolescents up to 18 years old
- full description of the clinical case/cases

The exclusion criteria were:

- reports not in English
- not pertinent to the field of investigation
- involving adults (> 18 years)
- not presenting the clinical description of the patient

To reduce errors and bias, two researchers independently analyzed titles and abstracts to exclude distinctly irrelevant articles. Finally, the eligibility of the articles was confirmed by evaluating the full text. Disagreements regarding inclusion/exclusion were settled by discussion between the researchers.

Afterwards, data were compiled in a Microsoft Excel spreadsheet to calculate frequencies and percentages of the problems.

## Results

### The case report

N.B, a 13-year-old female Moldovan patient, was admitted to Bambino Gesù Children Hospital, Rome, Italy, in August 2022 with a large retroperitoneal mass of unknown origin. She had neither family history of autoimmune or auto inflammatory diseases nor known comorbidities.

**Fig. 1:**
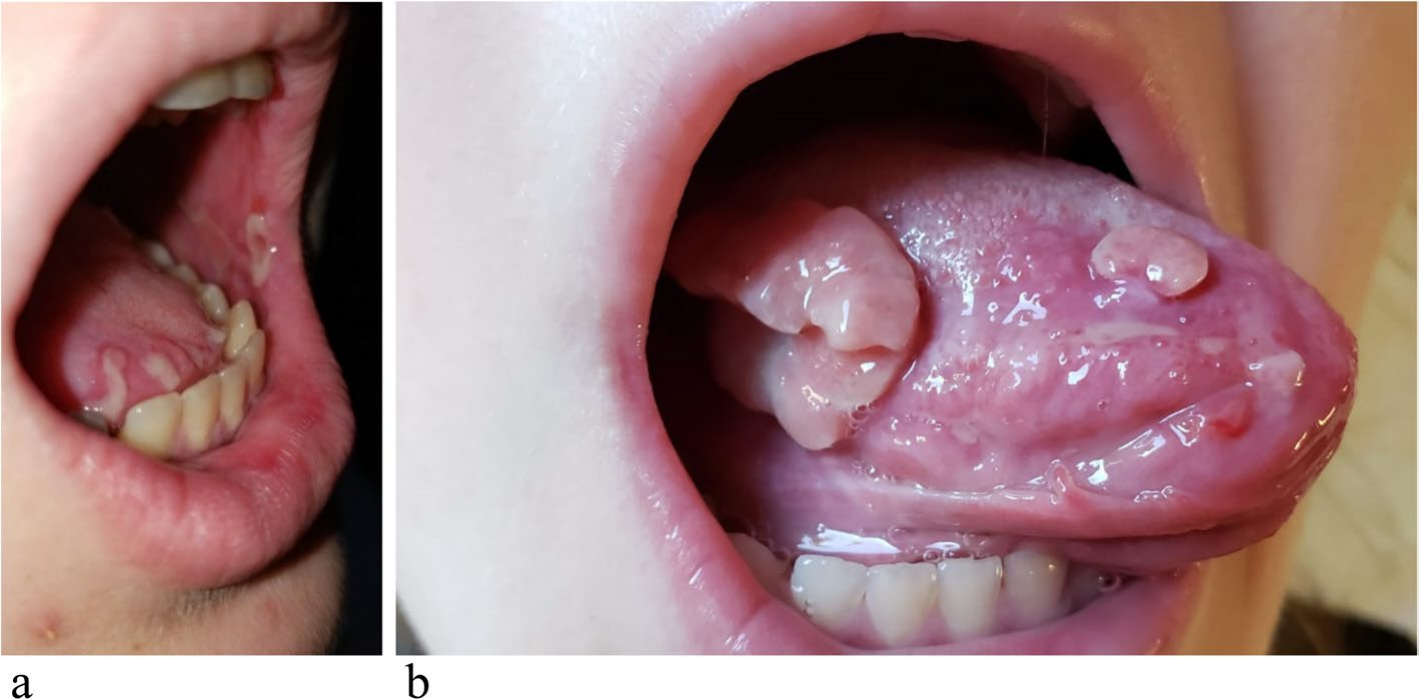
**a** Erosive and painful oral mucositis with erosions; **b** tongue pyogenic granulomas

She was a previously healthy girl with a mute personal history until April 2020, when she started presenting recurrent painful and erosive oral mucositis (Fig. 1a) and tongue neoformations in her mouth (Fig. 1b).

On July 2020, in Romania, three excised neoformations were histopathological analyzed, resulting compatible with pyogenic granulomas.

Despite different therapies, including chlorhexidine, metronidazole, dexamethasone, fluconazole, buccal symptoms were recurrent.

**Fig. 2:**
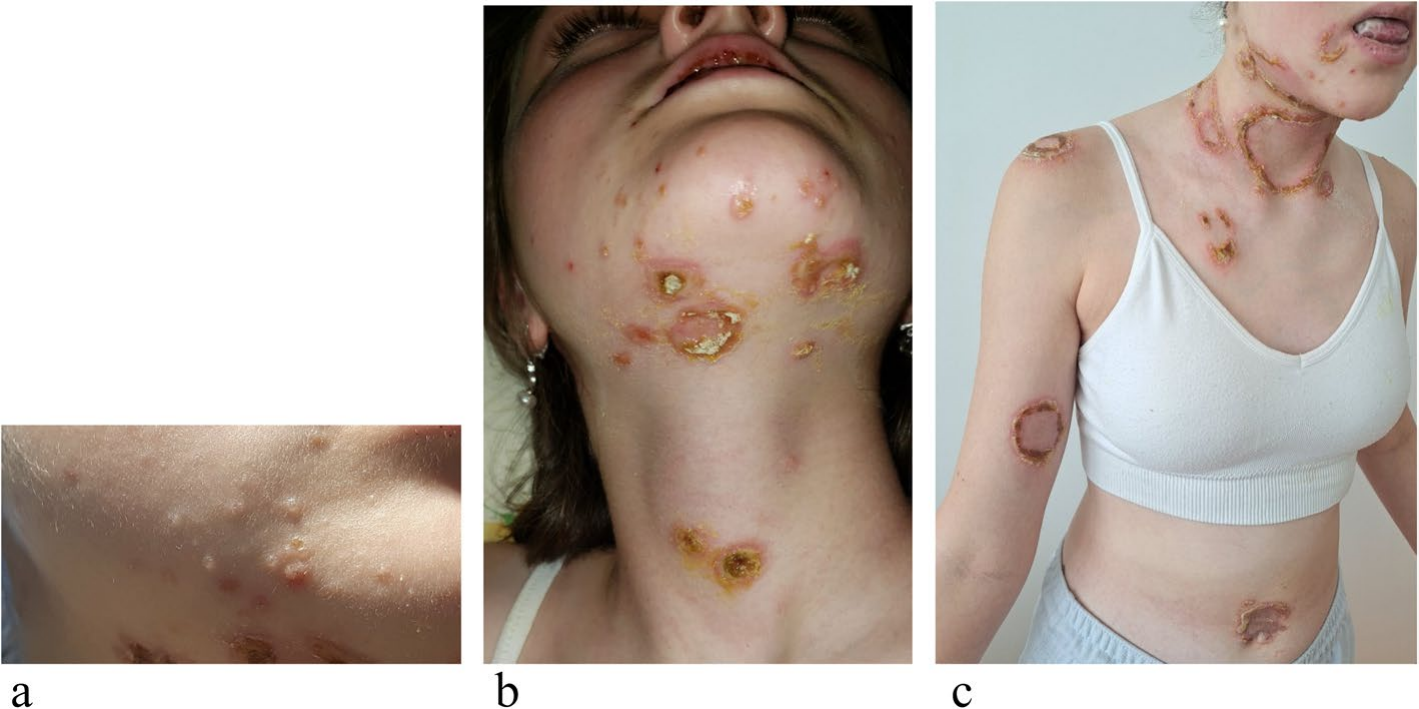
Skin vesicles on the face (**a**); targetoid crusted and erythematous lesions appeared on the face, neck, chest and shoulder (**b**, **c**)

In June 2022, new skin lesions appeared at chest, shoulder, perineum and face (Fig. 2a) in the form of vesicles coalescing in bullae and gradually transformed into targetoid, erythematous and crusted lesions (Fig. 2b,c).

In July 2022, she was hospitalized in Turkey with the suspect of pemphigus arisen from blood exam and skin biopsy. As the concern of PNP was raised, she was investigated for underlying malignancy and a PET-TC was performed with evidence of an overcaptive lesion in left iliac fossa (45 mm). An incisional biopsy just confirmed an aspecific "atypical lymphoid proliferation".

A few weeks later, the patient accessed an other Dermatological Hospital, where histological examination, serum direct immunofluorescence (DIF) and indirect immunofluorescence (IIF) of a new skin biopsy were performed.

Histological examination showed epidermal hyperplasia, spongiosis and lymphocytic infiltrate in the upper dermis. DIF revealed IgG and C3 intercellular deposition in the whole epidermis; IIF, with monkey esophagus epithelium, revealed epithelial cell surface attachment of IgG.

Anti-Desmoglein 1-Abs were 90 U/ml (> 20 positive), anti-Desmoglein 3-Abs were 180 U/ml (> 20 positive).

An abdominal ultrasound performed before doing biopsy of that lesion revealed the presence of the abdominal mass.

**Fig. 3:**
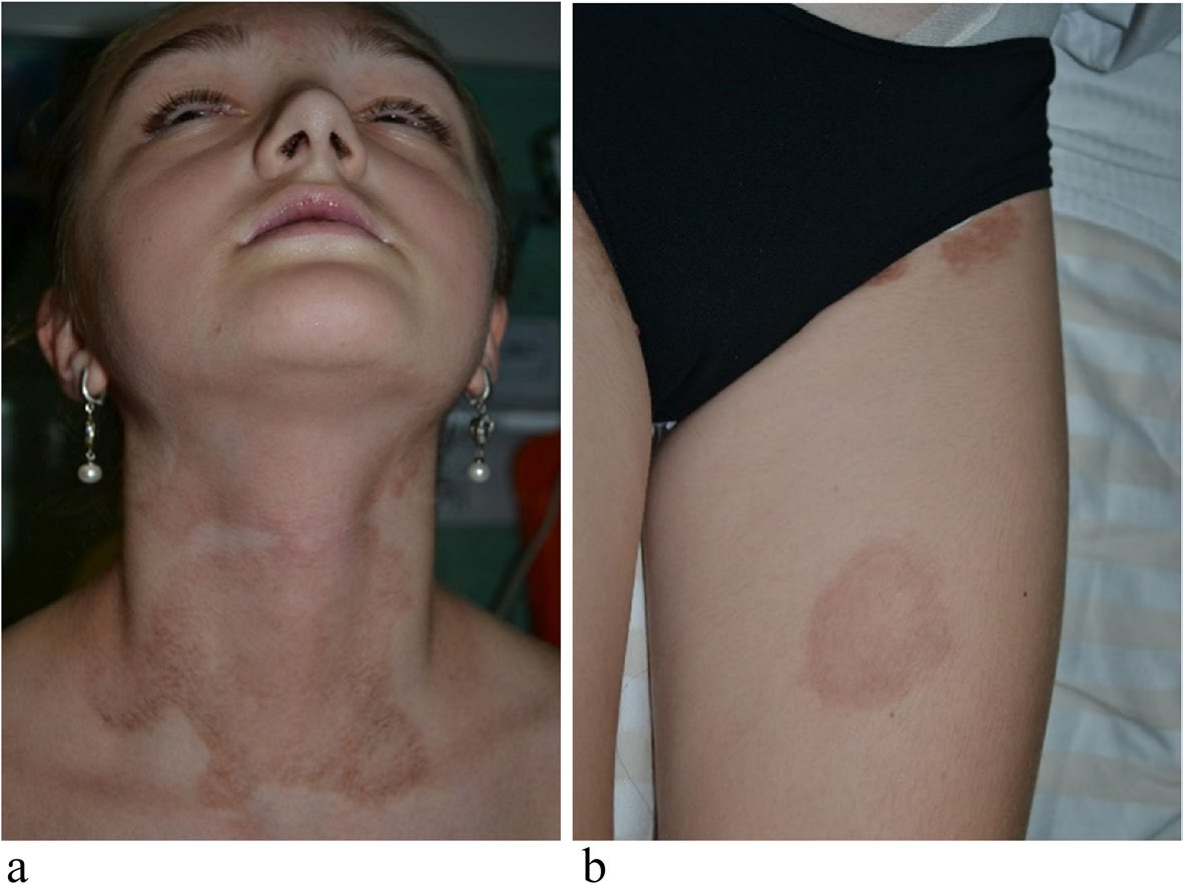
Hyperchromic annular macules, sequelae of previous erythematous lesions, on the neck (**a**) and the left thigh (**b**)

**Fig. 4:**
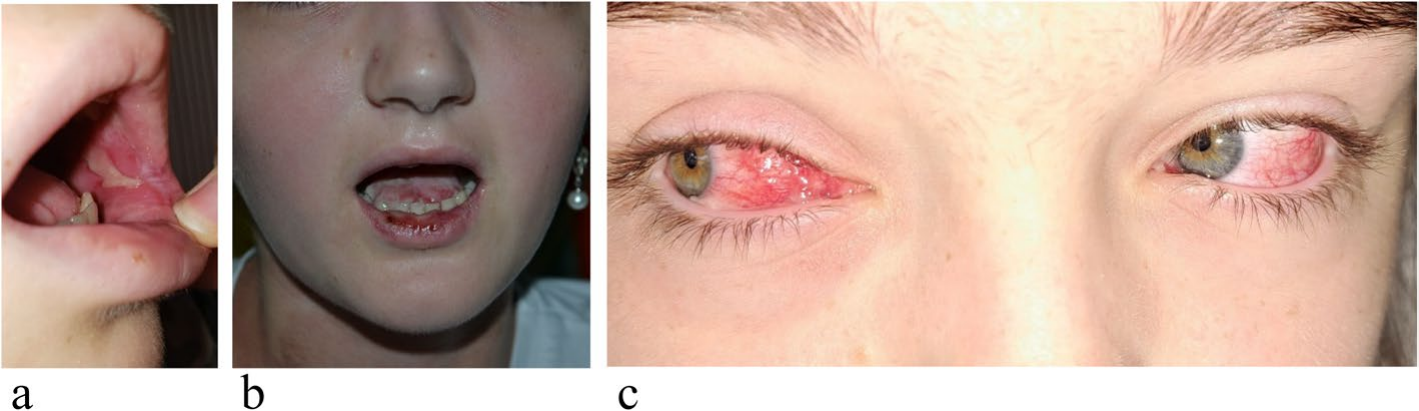
Recurrent erosive mucositis (**a**), with gingivitis and hemorrhagic crusts on the lips; bilateral conjunctival hyperemia (**c**)

To reach a comprehensive diagnosis, in August 2022, she was admitted to our hospital. She presented as an ill-looking patient with hyperchromic macules, sequelae of previous lesions, on the neck, back, on the chest and on the perineum (Fig. 3a,b). She also had severe mucositis, with gingivitis, extensive erosions on the tongue, hemorrhagic crusts on the lips, and bilateral conjunctival hyperemia (Fig. 4a-c).

An ultrasound scan of the abdomen confirmed an oval morphology solid tissue with irregular margins characterized by inhomogeneous echo structure in the left iliac fossa, associated with inhomogeneity of the adjacent adipose tissue.

An Magnetic resonance imaging better defined the polylobate neoformation, measuring approximately 10 cm × 5 cm × 5.6 cm, with marked contrast enhancement.

Laboratory tests, including, tumor markers, leukoscreening and tests for infectious diseases were normal. Patient's immune status was normal as well: lymphocyte subpopulations, immunoglobulin level, response to vaccination were normal.

**Fig. 5:**
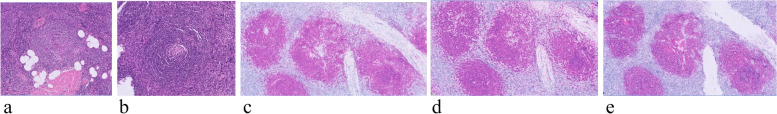
**A** Lymphoid follicle with atrophic germinal centre and numerous radially oriented penetrating hyalinized blood vessels (“lollipop” appereance). Note the perifollicular areas of sclerosis and the sclerotic blood vessels. **B** Lymphoid follicle with atrophic germinal centre and expanded mantle zone arranged in concentric rings (“onion skin” appearance). **C** CD20 immunostain shows many B follicles with atrophic germinal centres. **D** IgD immunostain shows the follicles mainly composed of mantle B-cells with a concentric arrangement. **E** CD21 immunostain shows the atrophic germinal centres mainly composed of hyperplastic follicular dendritic cells

A needle biopsy revealed a non-specific lymphoid tissue with fibro sclerotic changes, therefore an exploratory laparotomy was performed and the retroperitoneal mass was only partially excised because of difficulty of surgical access. Histopathological findings were compatible with hyaline vascular variant of Castleman disease. PET-scan demonstrated retroperitoneal lymphonodes (dm 4 × 3 × 6 cm, SUV 4,5) so it was considered a multicentric CD (Fig. 5a-e). Therefore, Rituximab was administered at a dose of 375 mg/m2/week for 4 consecutive weeks, by achieving an unsatisfactory response: PET-scan documented mild reduction of lymph nodes and oral erosions and conjunctival hyperemia were still observed.

For that, the patient was considered for therapy with Siltuximab, even if that is in indication for the treatment of adult patient affected by MCD. Actually, the disease is under control.

### Literature review

The search on the selected database has produced 118 results among articles and reviews. All the documents have been reviewed for relevance and eligibility. No duplications were identified.

According to PRISMA guidelines, of the identified items, all abstracts were analyzed, and 20 records were excluded as dealing with other topics, irrelevant to this review. Therefore, 98 were the records to be analyzed reading their full-length articles. All of them have been retrieved and assessed for eligibility, and then 57 were excluded because included adult population in their analyses, 25 had reported only generic information of patients, and 18 articles were not in English.

Finally, one relevant report cited in another study, was added to this research even if not originally included in Mesh research.

In conclusion, 17 were the records included in the revision [7–23]. 

**Fig. 6:**
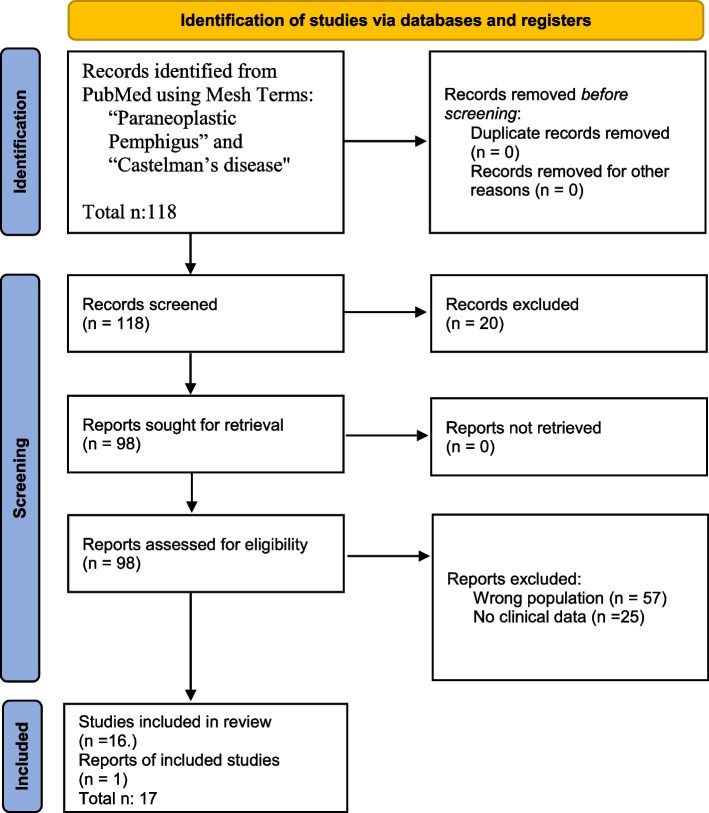
Flow chart of the selected process

Diagram 1 presents the flow chart of the selection process, adapted from PRISMA guideline (Fig. 6).

The included reports had been analyzed and the main highlights are presented in Table 1.

**Table 1:** Main findings of patients affected with PNP associated with CD reported in literature

Reference	Number of patients	Case number	Age (years) /sex	Personal history	Initial symptoms	Time to diagnosis	Mucosal manifestation	Skin manifestation	Systemic symptoms	Bronchiolitis obliterans
Index case	1	Index case	13/F	No	Mucosal (oral)	2 years	Oral Conjunctival	Blistering eruption	No	No
Shu‐Ping Han et al. [7]	1	1	13/F	No	Mucosal (oral)	6 months	Oral	Lichen planus	No	Yes
Mimouni et al. [8]	1	2	9/M	No	Mucosal (Oral, Conjunctival)	3 weeks	Oral Conjunctival	Blistering eruption Papular lichenoid rash Dystrophic nails	Dyspnea	Yes
Choh NA et al. [9]	1	3	16/M	No	Mucosal (oral) Skin Low grade fever	1 month	Oral	Lichenoid rash	Fever	Yes
Daneshpazhooh M et al. [10]	1	4	10/F	Resection of cervical UCD (HV) 5 years before	Mucosal (oral, conjunctival) Skin	2 months	Oral Conjunctival	Blistering eruption Papular lichenoid rash Dystrophic nails	Fever Dyspnea Cough	Yes
Jindal T et al. [11]	1	5	14/F	No	Mucosal (oral)	5 months	Oral	No	No	Yes
Koch LH et al. [12]	1	6	14/F	No	Mucosal (Oral, Conjunctival, Genital)	3 weeks	Oral Conjunctival Genital	Blistering eruption	No	Yes
Jing L et al. [13]	1	7	16/F	No	Mucosal (oral)	3 months	Oral Conjunctival Genital	Blistering eruption	No	No
Hung IJ et al. [14]	1	8	14/M	No	Mucosal (oral) Skin Cough	1 month	Oral Conjunctival Genital	Papular rash	Fever Cough	Yes
Lane JE et al. [15]	1	9	10/M	No	Mucosal (oral, conjunctival) Skin	2 months	Oral Conjunctival Genital	Papular rash Dystrophic nails	Fever Cough Fatigue Constipation Weight loss (no food intake)	Yes
Chin AC et al. [16]	1	10	14/M	No	Mucosal (Oral, Conjunctival, Genital)	3 months	Oral Conjunctival Genital	No	Dyspnea	Yes
Nousari HC et al. [17]	1	11	13/M	No	Mucosal (oral)	Not available	Oral Genital	Blistering eruption	No	Yes
Lemon MA et al. [18]	1	12	13/M	No	Epistaxis Mucosal (oral, conjunctival)	1.5 months	Oral Conjunctival Genital	Maculopapular eruption Swollen nail folds Nails shedding	Weight loss (no food intake)	No
Wang J et al. [19]	2	13 14	17/M 17/F	Not available Not available	Mucosal (oral) Mucosal (oral)	6 months 4 months	Oral Conjunctival Genital Oral Conjunctival Genital	Erythema multiform Lichen planus	Not available Not available	Yes Yes
Kumar S et al. [20]	1	15	15/M	No	Mucosal (oral) Abdominal pain	3 months	Oral	No	Abdominal pain Weight loss (no food intake)	No
Coulson IH et al. [21]	1	16	17/F	No	Mucosal (oral)	2 years	Oral Conjunctival Genital	Acral lichenoid eruption	Aseptic meningitis	Not available
Velasco et al. [22]	1	17	13/F	Not available	Mucosal (oral and conjunctival)	3 months	Oral Conjunctival	No	Thyroid carcinoma	Yes
Sathishkumar et al. [23]	2	18 19	8/F 8/M	Not available Not available	Mucosal Mucosal	Not available Not available	Oral Conjunctival Genital Oral Conjunctival	Pemphigus vulgaris Lichen planus Pemphigus vulgaris Lichen planus Papular rash	Not available Not available	No No

Reference	Case number	Form and Histological findings	Tumor localization	Tumor resection	Medication	outcome	Survival time
Index case	Index case	MCD (HV)	Retroperitoneal	No	Steroids Rituximab Siltuximab	Survive	Not available
Shu‐Ping Han et al. [7]	1	UCD (HV-SR)	Retroperitoneal (Pararenal)	Yes, complete	oral methylprednisolone rituximab tocilizumab plasmapheresis Immunoglobulin (2 g/kg)	Survive	12 months
Mimouni et al. [8]	2	UCD (MX)	Retroperitoneal	Yes, complete	Prednisone Steroid Radiotherapy Rituximab Daclizumab	Survive	Not available
Choh NA et al. [9]	3	UCD (HV)	Retroperitoneal	Yes, complete	Systemic and local steroids	Death (respiratory failure)	1 month
Daneshpazhooh M et al. [10]	4	UCD (HV)	Cervical	Yes, complete	Prednisone Azathioprine	Death (respiratory failure)	2 months
Jindal T et al. [11]	5	UCD (HV)	Chest (paratracheal area)	Yes, complete	Prednisone	Death (respiratory failure)	4 months
Koch LH et al. [12]	6	UCD (HV)	Retroperitoneal	Yes, complete	Prednisone Cyclophosphamide Thalidomide Tocilizumab	Survive	Not available
Jing L et al. [13]	7	UCD (HV)	Retroperitoneal	Yes, complete	Prednisone Plasmapheresis	Survive	Not available
Hung IJ et al. [14]	8	UCD (HV)	Chest (paratracheal region)	Yes, complete	Steroids	Death (respiratory failure)	7 months
Lane JE et al. [15]	9	UCD (HV)	Retroperitoneal	Yes, partial	Prednisone Immunoglobulin Cyclosporine Rituximab Daclizumab Radiotherapy	Death (respiratory failure)	15 months
Chin AC et al. [16]	10	UCD (HV)	Chest (Mediastinum)	Yes, complete	Prednisone Azathioprine Colchicine Thalidomide Embrel Cyclosporine Rapamycin Plasmapheresis Pirfenidone Living-related bilateral lung-lobe transplantation	Survive	Not available
Nousari HC et al. [17]	11	UCD (HV)	Chest (Mediastinum)	Yes, complete	Prednisone Cyclosporine Plasmapheresis	Death (respiratory failure)	30 months
Lemon MA et al. [18]	12	UCD (HV)	Chest (Mediastinum)	Yes, complete	Prednisone Cyclophosphamide Plasmapheresis	Survive	Not available
Wang J et al. [19]	13 14	17/M UCD (HV) 17/F UCD (HV)	Retroperitoneal Chest (Mediastinum)	Yes, complete Yes, complete	Not available Immunoglobulin	Not available Not available	Not available Not available
Kumar S et al. [20]	15	UCD	Extra peritoneal region of right hemipelvis	Yes, complete	Steroids	Survive	Not available
Coulson IH et al. [21]	16	UCD (MX)	Retroperitoneal	Yes, complete	Prednisone	Survive	Not available
Velasco et al. [22]	17	UCD (HV)	Chest (Mediastinum)	Yes, complete	Corticosteroids Rituximab Immunoglobulin Lung transplantation	Death	34 months
Sathishkumar et al. [23]	18 19	8/F UCD (HV) 8/M UCD (HV)	Retroperitoneal Cervical	Yes, complete Yes, complete	Prednisone Methotrexate Prednisone	Survive Survive	Not available Not available

*F* Female, *M* Male, *UCD (HV-SR)* Unicentric Castleman’s Disease (Hyaline-Vascular, Stroma Rich variant), *UCD (MX)* Unicentric Castleman’s Disease (Mixed variant)

### Patient demography

Twenty cases were included in this review (10 male and 10 female patients). The median age at diagnosis was 13.5 years (range from 9 to 17). In most cases, they were healthy until the onset of the disease. Only one patient had the history of a resection of cervical CD 5 years before.

### Clinical manifestations

In all cases, the initial manifestations were mucocutaneous lesions, especially oral lesions with poor response to conventional treatment.

Time from onset to diagnosis was on average 3 months (range from 3 weeks to 2 years).

The oral mucosal involment was exhibited in all patients, the conjunctival mucosa in 75% (15 of 20), and the genital mucosa in 55% (11 of 20).

Polymorphous skin rashes, including lichen planus, papules, blisters and erythema multiform, were noted in many patients (80%, 16 of 20). The lichenoid feature was the most frequently observed.

Some patients showed nail problems (3 patients with dystrophic nails, 1 patient with swollen nail folds and nails shedding).

Some patients experienced systemic symptoms (9 of 16): fever (25%, 4 of 16), dyspnoea (19%, 3 of 16), cough (19%, 3 of 16), and weight loss due to reduced intake of food secondary to oral pain (19%, 3 of 16), fatigue, constipation and abdominal pain. One patient had concomitant aseptic meningitis and one thyroid carcinoma.

PNP with BO was observed in 13 out of 19 patients (68%).

### Histological findings and localization of CD

In all cases, except for the index case, CD was found to be unicentric. One of them (case number 4) developed Castleman’s tumour recurrence after surgical resection.

Excluding one case (case number 14) where histologic pattern is not specified, from a histological point of view 19 cases were HV variants (89%, 17 of 19) and mixed type variants (11%, 2 of 19).

Main tumour localizations were retroperitoneal (50%, 10 of 20) and intrathoracic (45%, 9 of 20). Only one case was extra peritoneal in pelvis.

### Treatment

Almost all patients received corticosteroids with poor response. In UCD the mucocutaneous manifestations gradually resolved after tumour resection. All patients underwent complete surgical resection of the tumour, except one patient (case number 9) who underwent partial resection. Unfortunately, pulmonary involvement persisted also after tumour resection. Other drugs like azathioprine, methotrexate, cyclosporine, cyclophosphamide, thalidomide, immunoglobulin, rituximab, tocilizumab, daclizumab, plasmapheresis were used as adjuvant therapies for treating severe mucocutaneous lesions and BO. Two patients underwent lung transplantation.

In our case index, MCD was treated with rituximab and siltuximab, in agreement with the literature.

### Outcome

The outcome was recorded in 18 patients. Overall, 11 patients survived (61%) and 7 patients (39%) died in 1 month to 2.5 years after the diagnosis.

All survived patients underwent complete tumour resection, except our case index that is a multicentric form. Among the 11 survivors, 4 had BO (36%), 6 had not (55%) and one is not available. In the not survived group, all patients had BO and respiratory failure was the cause of death.

The mortality rate in the patients with and without pulmonary involvement was respectively 39% (7 of 18) and 0% (0 of 18), respectively (*P* = 0.035).

## Discussion and conclusions

In childhood, CD is the neoplasm most frequently associated with PNP [2]. The presence of PNP was identified as an independent unfavorable survival risk factor for patient with CD [24].

Our case report involved a previously health adolescent girl with refractory oral manifestations since two years before CD diagnosis.

Analyzing the literature, PNP onset is more frequent in otherwise healthy adolescence, without gender difference. In all patients, the first manifestation is oral ulcers refractory to conventional therapies. Skin involvement is present in almost all patients, especially as lichenoid rash.

Almost half of patients (56%) experienced systemic symptoms, in particular fever, dyspnoea, cough, weight loss, due to reduced intake of food secondary to oral pain. In our case report, no systemic symptoms had been referred.

From a histological point of view, CD is classified into three subtypes: hyaline vascular, plasma cell and mixed. In pediatric population is registered that the unicentric CD usually corresponds to the hyaline vascular variant, and the multicentric disease corresponds to the plasma cell variant. Stroma-rich hyaline-vascular CD appears to be a distinctive histological variant of the CD usually associated with paraneoplastic pemphigus [25–27]. In addition, our results are consistent with reported literature; in fact, almost all patients had unicentric HV CD (89%, 17 of 19).

It is unclear why CD is related to the pathogenesis of PNP. One hypothesis is that Castleman's tumors' proteins function as antigens and induce the production of autoantibodies that then cross-reacted with skin antigens from the plakin and cadherin family expressed in keratinocytes, particularly envoplakin (210 kD) and periplakin (190 kD). On the other side, secreted autoantibody from Castleman's tumours, which reacts against epidermal proteins, could be an essential factor in the pathogenesis of PNP [5, 6, 28, 29].

This is still to be clearly demonstrated as a few reports have shown paraneoplastic pemphigus with negative autoantibodies, suggesting the role of cytotoxic T-cells [30].

The increased risk of paraneoplastic pemphigus in stroma-rich hyaline vascular variant of CD can be explained by two possible mechanisms: the hyalinised vessels express plakin protein, which could incite antibody production against epidermal plakin; in a similar way, myoid cells could potentially act as antigen presenting cells activating immune response against plakin and desmoglein [29].

In our study main tumour, localizations were retroperitoneal and intrathoracic.

In literature the mainstay PNP treatment is the resection of the underlying neoplasm which lead to an improvement or remission of mucocutaneous lesions, along with reduction in antibody titers [31].

In our review, almost all patients underwent complete surgical resection of the tumour, except one patient and case index, who underwent partial resection. Most worrying in PNP associated with CD is BO, a diffuse segmental constrictive bronchiolitis. Its pathogenesis is not clear: in addition to autoantibody-mediated injury, CD8 + T-lymphocytes may have an important role in the progression of bronchiolitis. Pulmonary involvement persisted also after tumour resection and BO gradually progresses to respiratory failure and death [10, 11].

In our review, all patients received corticosteroids with poor response. Immunosuppressant, IVIG, rituximab, tocilizumab, daclizumab, plasmapheresis were also used like adjuvant therapies for treating severe mucocutaneous lesions and BO [7–23, 31–33]. Treatment of iMCD is challenging, and outcomes can be poor because no uniform treatment guidelines exist, few systematic studies have been conducted, and no agreed upon response criteria have been described.. The anti–interleukin-6 monoclonal antibody siltuximab (or tocilizumab, if siltuximab is not available) with or without corticosteroids is the preferred first-line therapy for iMCD [32].

The outcome was survival in 11 of 18 patients, death in 7 patients. BO seems an important prognostic factor: among the 11 survivors, 4 had BO (36%) and 6 (55%) did not, whereas in the mortality group, all patients had BO and respiratory failure was the cause of death.

Among survivors affected by BO two patient underwent lung transplantation with success, so lung transplantation must be considered in patient with BO [33].

## Conclusions

PNP should be always considered when investigating atypical mucocutaneous lesions such as pemphigus non-responding to conventional treatment. Screening for associated diseases may be mandatory in case of suspicious, in order to promptly obtain a diagnosis and start an adequate therapy. In childhood most patients have an unicentric CD that responds well to surgery; multicentric forms are rarely observed and require systemic therapy. In conclusion, treatment of CD in pediatric patients is not yet uniform and needs further studies and experimental trials.

## Data Availability

The Authors confirm that the data supporting the findings of this study are available at the authors ‘office.

## Abbreviations

PNP: Paraneoplastic pemphigus
CD: Castelman’s disease
UCD: Unicentric Castelman’s disease
MCD: Multicentric Castelman’s disease
DIF: Direct immunofluorescence
IIF: Indirect immunofluorescence
UCD (HV-SR): Unicentric Castleman’s Disease (Hyaline-Vascular, Stroma Rich variant)
UCD (MX): Unicentric Castleman’s Disease (Mixed variant)
